# The Effect of Genistein Supplementation on Cholesterol Oxidation Products and Fatty Acid Profiles in Serums of Rats with Breast Cancer

**DOI:** 10.3390/foods11040605

**Published:** 2022-02-20

**Authors:** Karolina Banyś, Agnieszka Stawarska, Rafał Wyrębiak, Wojciech Bielecki, Barbara Bobrowska-Korczak

**Affiliations:** 1 Department of Bromatology, Faculty of Pharmacy, Medical University of Warsaw, Banacha 1, 02-097 Warsaw, Poland; banyskarolina@gmail.com (K.B.); agnieszka.stawarska@wum.edu.pl (A.S.); 2 Department of Biomaterials Chemistry, Analytical Chemistry and Biomaterials, Faculty of Pharmacy Medica, University of Warsaw, Banacha 1, 02-097 Warsaw, Poland; rafal.wyrebiak@gmail.com; 3 Department of Pathology and Veterinary Diagnostics, Institute of Veterinary Medicine, Warsaw University of Live Sciences, Nowoursynowska 159c, 02-787 Warsaw, Poland; wojciech_bielecki@sggw.edu.pl

**Keywords:** genistein, cancer, nanoparticles, macroparticles

## Abstract

The aim of this study was to assess the effect of genistein on the level of cholesterol, oxysterols, and composition of fatty acids, as well as enzymatic activity of desaturases, in rats with breast cancer. The animals were supplemented with nano-, micro-, and macrogenistein. Rats were treated with 7,12-dimethylbenz[a]anthracene to induce mammary adenocarcinoma. In the case of animals supplemented with genistein, an increase in the intensity of the carcinogenesis process was observed. Genistein supplementation also affected the cholesterol and oxysterols levels, as well as the composition of fatty acids, in the serum of rats with neoplastic disease. Dietary supplementation with nanogenistein significantly increased the level of cholesterol (*p* = 0.02) and cholesterol oxidation products (*p* = 0.02), which may have significant impacts on cancer development and progression.

## 1. Introduction

Genistein is an isoflavone with variable biological activity [1,2]. The prophylactic and therapeutic effect of genistein on various types of neoplasms has been proven and confirmed in numerous clinical studies [3]. Genistein inhibits the activity of tyrosine kinases responsible for transmitting the signal of cell growth and topoisomerase II, a DNA stabilizing protein [4]. In this mechanism, genistein induces apoptosis. Additionally, genistein has been shown to cause cell cycle arrest. The described properties mean that genistein has cytotoxic properties on cancer cells, but it can also affect healthy cells the same way, causing mutations [4]. Both in vitro and in vivo studies have shown that genistein can stimulate the growth of some types of cancer, in particular hormone-dependent types of cancer, such as cancer of the cervix, breast, or ovary, which is due to its structure similar to 17-β-estradiol [5,6,7,8]. Interestingly, our previous study in rats showed that genistein may have different properties depending on the size of the molecule [9]. Nano-sized micronized particles have been shown to accelerate cancer growth. In the above study, supplementation of the animals’ diet with genistein resulted in increased levels of methylated derivatives in the urine of rats [9]. Nanometric particles differ from the standard-sized material, despite having the same composition because, below a certain size, quantum effects significantly affect the physicochemical properties and behavior of the particle. The purpose of creating nanomaterials is to show new or improved physical, chemical, and biological properties of already known materials. For this reason, nanoparticle technology is used in medicine for the diagnosis and treatment of patients. However, in our earlier study, the reduction of genistein particles to nanosized particles had the opposite effect to the desired therapeutic effect [9].

Fatty acids are biologically active compounds and play key roles in various metabolic pathways in both healthy and diseased organisms. They influence metabolism both at the tissue and cellular level, functioning, response, hormone signaling, etc. [10,11]. Over the years, clinical and epidemiological studies have shown that some fatty acids may have negative effects on the human body, causing an increased risk of cardiovascular disease, neurological diseases, cancer, nonalcoholic fatty liver disease, etc. [12]. Saturated fatty acids (SFAs) are very important for the body, as they are part of the phospholipid structure of cell membranes. On the other hand, numerous studies have shown that diets rich in these nutrients increase the risk of cardiovascular disease, inflammation, type 2 diabetes, cancer, and kidney disease. In the neoplastic tissues of the lung, prostate, breast, ovary, endometrium, colon, and bladder, compared with normal tissues, increased levels of SFAs have been confirmed [10,11,12]. Studies have shown that replacing saturated fatty acids with unsaturated acids significantly lowers LDL and improves lipid metabolism while reducing the risk of atherosclerosis and cardiovascular disease. Unsaturated acids also appear to have protective effects on the entire nervous system and seem to prevent certain types of cancer. N-6 and n-3 belonging to polyunsaturated fatty acids (PUFAs) are involved in the regulation of many important physiological and pathological processes, such as oxidative stress, inflammation, skin lesions, asthma, nervous system disorders, or cancer formation [9,10,11]. PUFAs are believed to exert their effects either indirectly, through various metabolites, or directly. The number of fatty acids in our body may be influenced by many factors, such as our daily diet, endocrine system, and the activity of PUFA-converting enzymes (desaturases, elongases) [9,10,11]. The role of fatty acids in cancer risk and development is becoming more and more accepted since, aside from their principal roles as structural components of the membrane matrix, they are important secondary messengers and can also serve as fuel sources for energy production [13]. Either high- or low-dose genistein treatment can affect metabolic disorders and inflammatory processes by changes in fatty acids composition and metabolism [14,15]. The results indicated that genistein supplementation can upregulate the expression of genes related to fatty acid synthesis. Genistein can activate peroxisome proliferator-activated receptor (PPARα) and PPARγ simultaneously and promote fatty acids β-oxidation. It was found that genistein upregulated the fatty acid transporters [14,15].

Oxysterols, or compounds derived from cholesterol oxidation, are bioactive lipids involved in many cellular mechanisms underlying human pathophysiology. These compounds contribute to the intensification of inflammatory processes in the human body. They induce the expression and enhance the synthesis of pro-inflammatory cytokines, including tumor necrosis factor (TNF-α), the chemotactic monocyte protein-1 (MCP-1), chemokines, interleukins 1β (IL-1β) and 6 (IL-6), adhesion molecules, and activating phagocytes. They are also responsible for the activation of cytokine-dependent inflammatory processes that favor the development of many disease entities [16,17,18]. Clinical trials have proven their influence on the pathogenesis of type 2 diabetes, atherosclerosis, and neurodegenerative diseases [16,17,18]. Many scientific studies clearly show that both cholesterol and oxysterols have important roles in malignant neoplasms such as prostate, colon, and breast cancer [19]. For example, oxysterols produced by osteoblast-like MG63CM cells promote the migration of MCF7 and MDA-MB-231 breast cancer cells, which may indicate that these compounds are important navigators for the stimulation and formation of bone metastases in breast cancer patients [19]. The use of the level of a given oxysterol in the body to assess the risk of a given disease seems promising and worth investigating in further studies.

The aim of this study was to evaluate the effect of nano-, micro-, and macrogenistein on the level of cholesterol oxidation products and fatty acids profile in serum of rats with 7,12-dimethylbenz[a]anthracene-induced breast cancer (*adenocarcinoma*). Therefore, with this study, we aimed to clarify a molecular mechanism through which genistein supplementation affects the process of carcinogenesis by changes in the level of cholesterol and its derivatives and changes in the fatty acid composition and their metabolism.

## 2. Materials and Methods

### 2.1. Preparation of Genistein Micro- and Nanoparticles

The following compounds were purchased to carry out the micronization process: poly(vinyl acetate) (PVA), purity > 98.0%, Tokyo Chemical Industries (Portland, OR, USA); genistein, purity > 98.0%, Tokyo Chemical Industries (Portland, OR, USA); ethyl acetate was purchased from Avantor Performance Materials Poland S.A. (Gliwice, Poland).

The description of the procedure for obtaining genistein in nano- and microforms was presented in the publication of Banyś et al. [9]. Genistein in macroform was used as received. In order to study the mean size of genistein particles and their zeta potential, the dynamic light scattering (DLS) method was used. A Zetasizer Nano ZS analyzer (Malvern Instruments, Westborough, MA, USA) equipped with a red laser, with a wavelength of 633 nm and a scattering angle of 173° at 25 °C, was used for the tests. The sample was suspended in distilled water (zeta potential measurement) or in aqueous glycerol (particle size measurement). The obtained test results are presented in Table 1.

### 2.2. Laboratory Animals

This study was approved by the Ethics Committee (Decision No. 645/2018). The experimental animal was female Sprague Dawley rats (*n* = 32) obtained from the Animal Laboratory of the Department of General and Experimental Pathology, Medical University of Warsaw. All rats were fed a standard Labofeed H diet (Labofeed H, Żurawia 19, 89-240 Kcynia, Poland) and water ad libitum. They were placed in an environmentally controlled room with a 12 h light–dark cycle and a temperature of 22 °C. The rats, in accordance with the assumptions of the study, were divided into the following four experimental groups:

- A control group in which the animals were fed with a standard diet, without supplementation (they received 0.4 mL of water via a gavage);
- A group that received genistein nanoparticles (92 ± 41 nm) in dose 0.1 mg/mL, i.e., 0.2 mg/kg bw;
- A group that received genistein microparticles (587 ± 83) in dose 0.1 mg/mL, i.e., 0.2 mg/kg bw;
- A group that received genistein macromolecules in dose 0.1 mg/mL, i.e., 0.2 mg/kg bw.

Genistein, regardless of particle size, was suspended in 0.4 mL of water and administered to the animal by gavage from day 40 to week 20 of age. The polyphenol dose was calculated from the mean human daily consumption (extrapolated to the body weight of the rats).

To induce breast cancer (adenocarcinoma), rats were treated twice with DMBA (7,12-dimethyl-1,2-benz (a) anthracene; Sigma-Aldrich, St. Louis, MO, USA) which was suspended in rapeseed oil (via gavage). The first administration of DMBA was at 60 days of the animals’ age at a dose of 80 mg/kg body weight, and the next on day 90 at a dose of 40 mg/kg body weight. From 7 weeks of age, the animals were weighed weekly. In addition, rats were palpated daily to detect the appearance of tumors and to monitor for any specific disorders such as loss of appetite, hiding, and lethargy [9].

### 2.3. Histopathological Analysis of Tumors

Histopathological examination of the rat tumors was performed using the BX43 Olympus research microscope. The collected materials were previously stained with hematoxylin and eosin. Mitoses were counted at an objective magnification of 40× in randomly selected tumor preparations in 15 fields of view [9].

### 2.4. Determination of Fatty Acids, Cholesterol, and Its Oxidized Derivatives in Serum of Rats

The content of fatty acids, cholesterol, and its oxidized derivatives in rats’ serum was performed using a gas chromatograph with time-of-flight mass spectrometry (GC–TOFMS) (Pegasus^®^ BT, LECO Corporation, St. Joseph, MI, USA). The detailed procedures of using these methods were described in a previous publication [20]. The peroxidation index (PI) of the experimental groups was determined on the basis of the serum FA composition [21].

### 2.5. Estimation of Desaturases Activity

Desaturase activities were calculated as product/precursor ratios of individual fatty acids in serum [22]. In this study, desaturase activities were estimated as follows: the value of the D5D index was calculated as the ratio of the arachidonic acid concentration (AA, C20:4, n-6) to the concentration of dihomo-γ-linolenic acid (DGLA, C20:3, n-6); the value of the D6D index was calculated as the ratio of the concentration of γ-linolenic acid (GLA, C18:3, n-6) to the concentration of linoleic acid (LA, C18:2, n-6).

### 2.6. Statistical Analysis

Group relationships and differences were analyzed using one-way ANOVA (α = 0.05) with Tukey’s post hoc test (α = 0.05). The results are presented as mean value ± standard deviation.

## 3. Results

### 3.1. Histopathological Examination of Tumors after Treatment with Genistein

**Figure 1:**
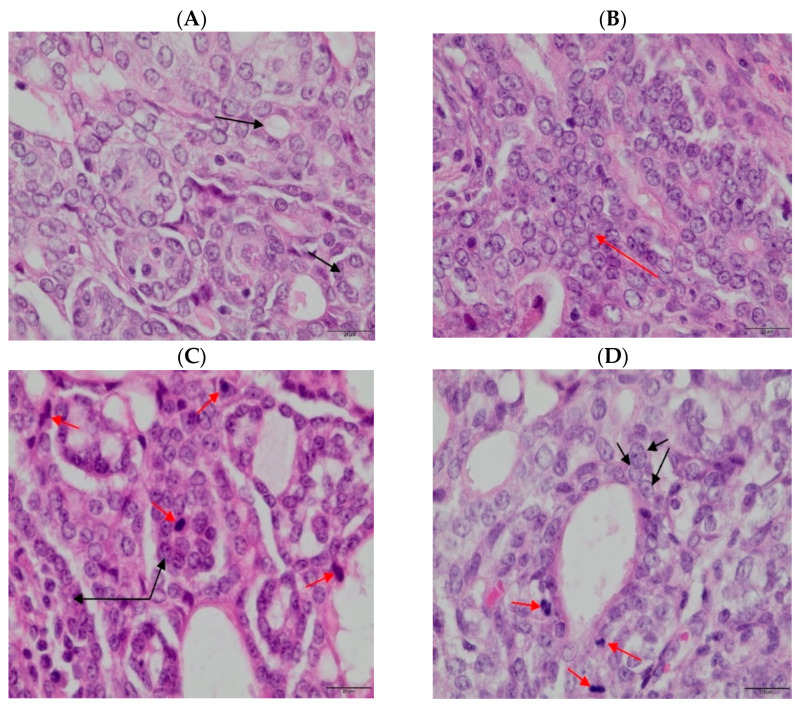
Biopsy images of tumor samples with 40× magnification; hematoxylin–eosin staining was applied. DMBA (7,12-dimethylbenz[a]anthracene)-induced tumor in the control group and macroparticles supplementation—II grade adenocarcinoma (**A**,**B**) and in the group with the nano- and microparticles supplementation—III grade adenocarcinoma (**C**,**D**); red arrows indicate mitosis, and black bands indicate tumor cells).

Moderately differentiated tumor cells, characteristic of grade 2 adenocarcinoma, were found in both the control group and the group supplemented with genistein macroparticles. In contrast, in the groups supplemented with genistein nano- and microparticles, grade 3 tumors were found (Table 2, Figure 1).

Carcinogenesis induced by DMBA administration to animals independent of diet (standard; supplemented with macro-, micro-, and nanoparticles) was not inhibited (Table 2, Figure 1). However, in the group supplemented with nanogenistein, the tumor cells multiplied faster because the first tumors in these groups of rats appeared 2 weeks earlier (14 weeks) than in animals from the control group and 3 weeks earlier than in the groups supplemented with macro and microgenistein. In animals supplemented with microgenistein, the incidence of tumors was 88%, and the number of tumors per rat ranged from 0 to 3. In other groups of animals, the carcinogenesis efficacy was 100%, and the number of tumors, depending on the groups of animals tested, ranged from 1 to 9 (Table 2). However, the mean weight of the tumors in the animals receiving microgenistein (1.99 ± 1.75) (20 weeks) was significantly higher than the weight of the tumors in the control animals (without supplementation) (0.93 ± 1.34). The lowest mean number of mitoses in the field of view (1.79 ± 1.25) was observed in the group of rats fed the diet without supplementation. On the other hand, in the groups of rats supplemented with macro-, micro-, and nanogenistein, the mean numbers of mitoses were higher and amounted to 4.46 ± 2.38, 7.33 ± 1.57, and 5.82 ± 1.57, respectively. The above results indicate that in animals supplemented with genistein, there were increased levels of proliferation of neoplastic cells.

### 3.2. The Rats’ Body Weight

**Figure 2:**
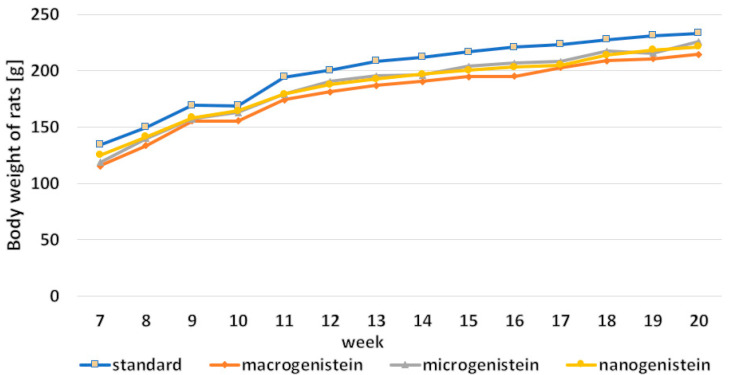
The rats’ body weight (g) in particular weeks of the experiment.

The animals’ body weight measurements in particular weeks of the experiment are presented in Figure 2. There were no statistically significant differences between body weight measurements depending on supplementation (Figure 2, Table 3). No adverse effects or poor health of animals were observed.

### 3.3. Determination of Cholesterol and Cholesterol Oxidation Products (Oxysterols)

**Figure 3:**
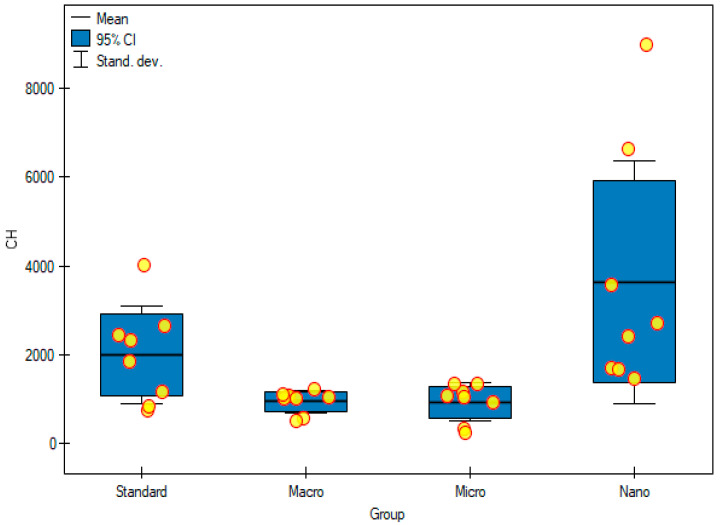
Content of cholesterol in experimental groups. Standard—control group; Macro—a group of rats receiving genistein in macromolecules; Micro—group of rats receiving genistein in microparticles; Nano—a group of rats receiving genistein in nanoparticles; CH—cholesterol.

**Figure 4:**
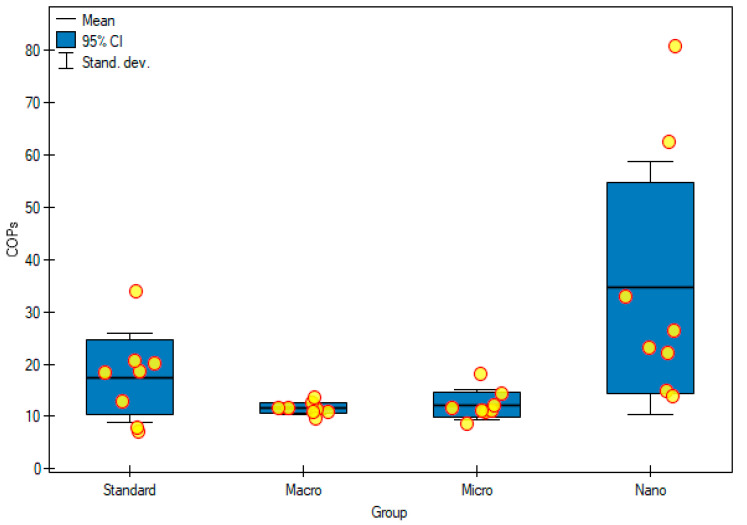
Content cholesterol oxidation products in experimental groups. Standard—control group; Macro—a group of rats receiving genistein in macromolecules; Micro—group of rats receiving genistein in microparticles; Nano—a group of rats receiving genistein in nanoparticles, COPs—cholesterol oxidation products.

Upon analyzing the above results, it was observed that the highest cholesterol concentration was in animals supplemented with nanogenistein (3643 ± 2727 μg/mL) and statistically significantly different from the group supplemented with macrogenistein (949 ± 259 μg/mL) and microgenistein (940 ± 425 μg/mL) (*p* = 0.0213) (Table 4, Figure 3). Moreover, the results clearly showed that animal nutrition with nanogenistein significantly increased the concentration of 7K-Ch (8.04 ± 5.05 μg/mL, *p* = 0.012) and 7α-OH-Ch (3.57 ± 2.68 μg/mL, *p* = 0.017) in serum, compared with that of groups of rats supplemented appropriately macrogenistein (7K-Ch (2.64 ± 0.68 μg/mL); 7α-OH-Ch (1.10 ± 0.55 μg/mL)) and microgenistein (7K-Ch (3.05 ± 0.90 μg/mL); 7α-OH-Ch (0.88 ± 0.14 μg/mL)). On the other hand, in the case of 5.6βE-Ch, the concentration was the highest in the group of rats with nanogenistein (16.65 ± 11.99 μg/mL), and it differed statistically significantly from the other studied groups (*p* = 0.014). The sum of cholesterol oxidation products was the highest in the group of rats fed with nanogenistein (34.59 ± 24.16 μL/mL) and significantly higher (*p* = 0.02) than that in the groups of rats fed with macrogenistein (11.55 ± 1.18 μg/mL) and microgenistein (12.23 ± 2.87 μg/mL) (Table 4, Figure 4).

### 3.4. Fatty Acids Content in Rats’ Serum

The following fatty acids dominated in the blood serum of the studied animals: palmitic (C16:0), stearic (C18:0), oleic (OL, C18:1 n-9), linoleic (LA, C18:2, n-6), and arachidonic (AA, C20:4 n-6), which had the largest share in the total fatty acid content (Table 5).

**Figure 5:**
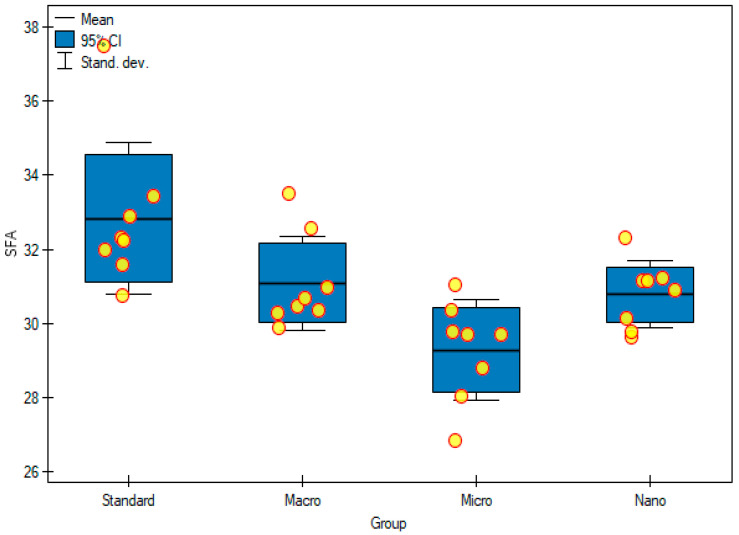
Saturated fatty acids profile (%) in experimental groups. Standard—control group; Macro—a group of rats receiving genistein in macromolecules; Micro—a group of rats receiving genistein in microparticles; Nano—a group of rats receiving genistein in nanoparticles; SFAs—saturated fatty acids.

The content of saturated fatty acids (SFAs), relative to all fatty acids, ranged from 29% to 33% (Table 6, Figure 5). It was observed that, in animals fed with nanogenistein and microgenistein, the concentrations of pentadecanoic acid (C15: 0) (23.64 ± 4.96 μg/mL; 20.39 ± 5.09 μg/mL) and heptadecanoic acid (C17:0) (23.55 ± 4.60 μg/mL; 21.97 ± 3.64 μg/mL) increased significantly, compared with those of the control group (*p* = 0.0004; *p* = 0.0015) (Table 5). Interestingly, no statistical differences were found between the groups when assessing the sum of all SFAs, but there was an upward trend in rats in the group with nanogenistein and microgenistein (Table 5). Nanogenistein (30.78 ± 0.89%) and microgenistein (29.28 ± 1.35%) influenced the percentage of SFAs in all fatty acids and were appropriately lower than in rats fed with a standard diet (32.83 ± 2.04%) (*p* = 0.0005) (Table 6, Figure 5).

**Figure 6:**
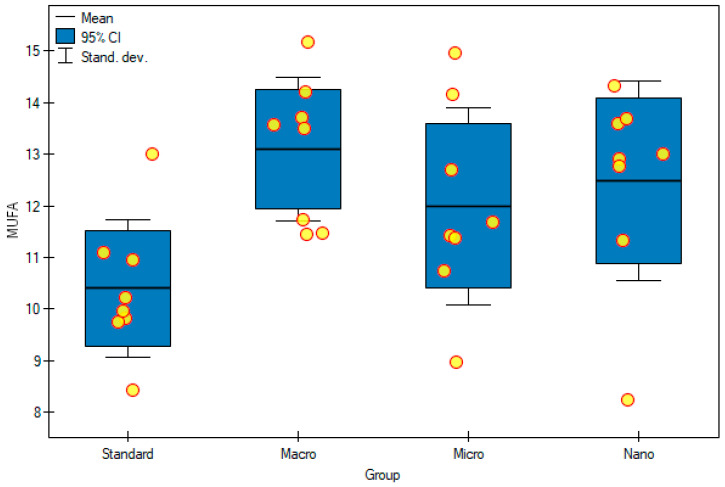
Monounsaturated fatty acid (%) in experimental groups. Standard—control group; Macro—a group of rats receiving genistein in macromolecules; Micro—a group of rats receiving genistein in microparticles; Nano—a group of rats receiving genistein in nanoparticles; MUFA—monounsaturated fatty acid.

**Figure 7:**
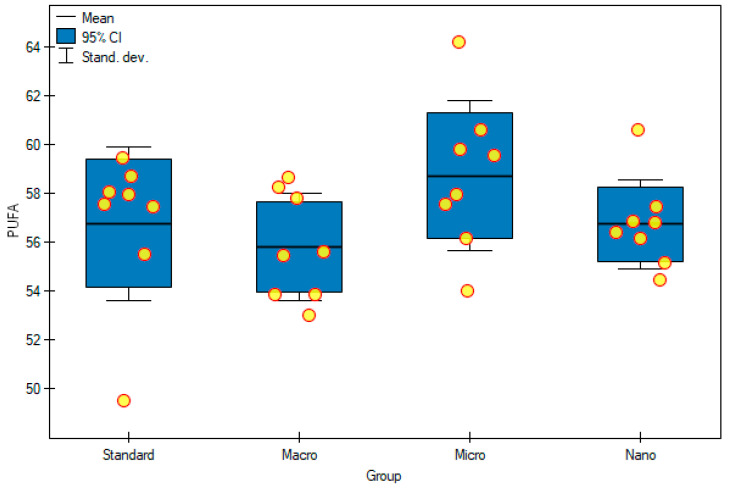
Polyunsaturated fatty acid (%) in experimental groups. Standard—control group; Macro—a group of rats receiving genistein in macromolecules; Micro—a group of rats receiving genistein in microparticles; Nano—a group of rats receiving genistein in nanoparticles; PUFA—polyunsaturated fatty acid.

The content of monounsaturated fatty acids (MUFAs), among all fatty acids, was lower than that of SFAs and ranged from 10% to 13% (Table 6, Figure 6). When assessing this group of fatty acids, a very similar tendency was observed to the case of SFAs. Rats that were fed nanogenistein and microgenistein had higher concentrations of MUFAs, although not statistically significant, than rats fed with a standard diet (without supplementation) (Table 5). Moreover, nanogenistein and microgenistein statistically increased the content of hexadecenoic acid (C16:1 n-9, *p* = 0.0003), palmitoleic acid (C16:1 n-7, *p* = 0.0013) and vaccenic acid (C18:1 n-11, *p* = 0.0047). In rats fed the standard diet (10.40 ± 1.33%), the percentage of all MUFAs was significantly lower than that in the group supplemented with macrogenistein (13.10 ± 1.39%) (Table 6, Figure 7). When analyzing the results for polyunsaturated fatty acids (PUFAs), it had the highest concentration of all fatty acids, from 56% to 59%, which is related to the fact that, in this study, as many as seven different PUFA, four MUFA, and six FSA concentrations were measured (Table 6, Figure 7). Interestingly, in most cases, apart from dihomo-γ-linolenic acid (C20:3 n-6 DGLA), the concentration of individual PUFAs, as well as their sum, statistically significantly increased in rats fed with nanogenistein or microgenistein (Table 5). In the case of rats that received microgenistein and nanogenistein, the level of arachidonic acid (1226 ± 197 μg/mL; 1114 ± 207 μg/mL, respectively) was higher than that in the group fed with macrogenistein (824 ± 168 μg/mL). A similar correlation was observed in the case of ALA (*p* = 0.015) and EPA (*p* = 0.024) acids. In the case of LA (*p* = 0.023) and GLA (*p* = 0.0001) acids, a statistically significant increase in the concentration of these acids was also observed in rats fed with nanogenistein and microgenistein, compared with those of the rats fed the standard diet (Table 5). Moreover, the ratio of MUFAs + PUFAs/SFAs was determined. We observed that the supplementation of rats with microgenistein and nanogenistein significantly increased the index, compared with the control group (*p* = 0.0003) (Table 6).

### 3.5. Desaturase Activity Indicators (D6D and D5D)

**Figure 8:**
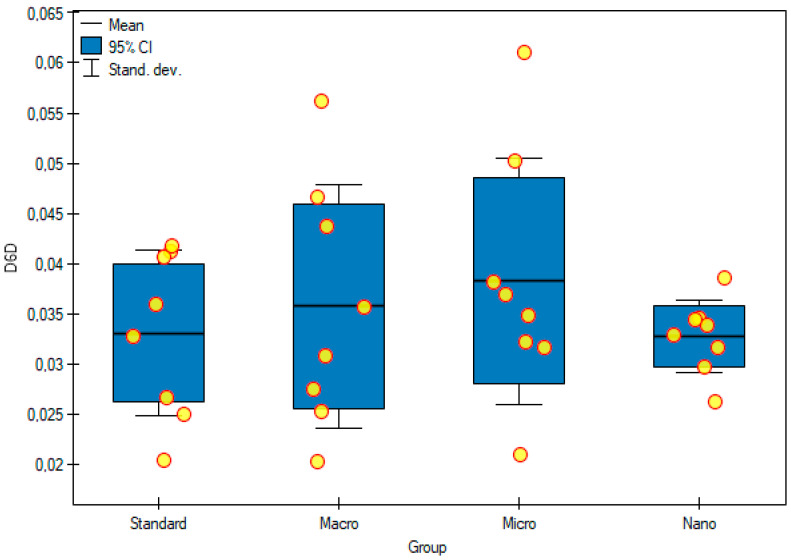
D6D activity in the serum of experimental groups. Standard—control group; Macro—a group of rats receiving genistein in macromolecules; Micro—a group of rats receiving genistein in microparticles; Nano—a group of rats receiving genistein in nanoparticles; D6D—Δ6-desaturase index. Not significant.

**Figure 9:**
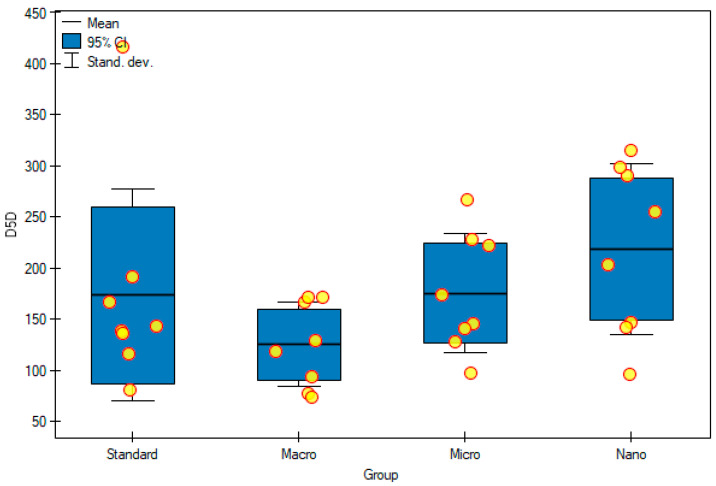
D5D activity in the serum of experimental groups. Standard—control group; Macro—a group of rats receiving genistein in macromolecules; Micro—a group of rats receiving genistein in microparticles; Nano—a group of rats receiving genistein in nanoparticles; D5D—Δ5-desaturase index. Not significant.

The ∆6-desaturase activity index (D6D) was calculated as the ratio of GLA/LA concentrations and the ∆5-desaturase activity index (D5D) as the ratio of AA/DGLA concentrations. The results obtained in the group of animals supplemented with genistein nanoparticles showed the lowest D6D activity ((32.74 ± 3.64) × 10-3), compared with that of the control group ((33.06 ± 8.22) × 10-3) and, at the same time, the highest D5D activity (218 ± 83) among all groups tested. Supplementation of animals with genistein macromolecules decreased the activity of D5D in the serum of rats (125 ± 41), compared with that of the control group (173 ± 103), but these differences were not statistically significant (Figure 8 and Figure 9).

## 4. Discussion

Soy is a common vegetable component of the diet of many people around the world. This plant is rich in isoflavonoids, including genistein, which is chemically similar to 17-β-estradiol, which binds to the estrogen receptor and shows a higher affinity for ER-β than for ER-α. The results of clinical trials and observations show that the effect of genistein is inconclusive—some of them show its protective effect and others a harmful effect. Our previous study conducted on rats proved that genistein, depending on the size of its molecule, modifies the development of neoplasms, and an important role in this process may be played, among others, by changes at the epigenetic level [9]. The next goal of our research was to evaluate the effect of nano-, micro-, and macrogenistein on the levels of oxysterols, fatty acids, and activity of desaturases in the serum of rats with breast cancer.

Cholesterol metabolism produces the necessary components of the cell membrane as well as metabolites that have various biological functions. During carcinogenesis, the signals of the internal and external cells reprogram the metabolism of cholesterol and consequently promote the formation of tumors. In this complex process, cells deregulate cholesterol homeostasis, causing intracellular cholesterol accumulation, which is required to maintain their high growth rate. Cancer cells increase the level of cholesterol, facilitating the biosynthesis and metabolism of lipids, and metabolites derived from cholesterol support the progression of cancer and suppress the immune response [23,24]. In our study, the concentration of cholesterol was the highest in the group of animals fed with nanogenistein (*p* = 0.02), which may indicate an intense proliferation of tumor cells in this group of rats. Elevated serum cholesterol levels are associated with increased incidence and recurrence of prostate cancer, while inhibition of its biosynthesis by using statins reduces colorectal, breast, and endometrial cancer mortality [12]. Oxysterols are products of the oxidation of cholesterol derivatives, both exogenous and endogenous. Over the years, they have been associated with many pathologies, including cancer and neurodegenerative diseases. It has been shown that, in addition to their role in carcinogenesis, migration, proliferation, apoptosis, and many signaling pathways, they also affect cancer therapy. They are gaining increasing interest as biomarkers in cancer treatment and in patient stratification to optimize therapy because differences in blood oxysterol levels in cancer patients at different stages, or compared with healthy patients, have been shown [25,26]. In line with the results obtained in other studies, it was found that 7-ketocholesterol, 7β-hydroxycholesterol, and 5β, 6β-epoxycholesterol are the main COPs [27]. The group of rats fed with nanogenistein was found to have the highest total COP concentration (*p* = 0.02). Genistein in this form showed an increased potential for inducing oxidative stress, which may result in a higher total COP content. It has been known for a long time that some oxysterols (7-K-Ch, 7α-hydroxycholesterol (7α-OH-Ch), 7β-OH-Ch, 5α6α-epoxycholesterol, 5,6βE-Ch, 25-hydroxycholesterol) have strong pro-inflammatory effects, and the degree of the inflammation contributes to the development of many disease entities. An increased risk of cancer, including breast cancer, is associated with high COP levels [28,29]. Interestingly, one study showed that the addition of 7K-Ch in breast cancer cell lines treated with doxorubicin decreased its cytotoxicity, thus reducing its effectiveness [24]. Another study by Kloudova-Spalenkova [30] showed that 7α-OH-Ch and 5,6βE-Ch had an effect on the size of neoplastic cells in breast cancer, and their concentration was higher in patients with advanced cancer or with larger tumors [30]. In our study, a significantly increased content of 7K-Ch, 7α-OH-Ch, 5,6βE-Ch in the serum of rats supplemented with nanogenistein was observed. This confirms that genistein, depending on the particle size, influences the biological activity and proliferation of tumor cells in rats with DMBA-induced breast cancer in vivo.

In the present study, we also tried to estimate if genistein supplementation affects serum fatty acids profile in pathological conditions caused by cancer. Under physiological conditions, for β-oxidation, saturated fatty acids are transferred to the mitochondria and then secreted into the blood plasma in the form of very low density lipoprotein. Unnecessary SFAs generate lipotoxic intermediates such as diacylglycerol and stimulate a number of cascade reactions. By binding to the Toll-like receptor 4, they induce effects such as activation of the pro-inflammatory nuclear factor κB (NF-κB) and exacerbation of mitochondrial dysfunction [31,32]. This leads to an increased synthesis of pro-inflammatory cytokines (interleukin 6, tumor necrosis factor α) and the biosynthesis of pro-inflammatory eicosanoids from arachidonic acid (prostaglandin E2). However, an increasing number of scientific reports point to the beneficial effect of odd saturated fatty acids on human health [31,32]. The results of our study show that genistein does not statistically significantly affect the total concentration of saturated fatty acids. However, when analyzing individual fatty acids in this group, a statistically significant increase in odd pentadecanoic (C15:0) and heptadecanoic (C17:0) fatty acids was observed in the group of rats supplemented with nano- and microgenistein. Interestingly, the research that Bao To described in 2020 [33] clearly indicates that both of these fatty acids showed cytotoxic effects on MCF-7 and MCF-7/SC cells. Moreover, pentadecanoic acid decreased MCF-7/SC hardness and suppressed the migratory and invasive capacity of MCF-7/SC, inhibited JAK2/STAT3 signaling induced by interleukin-6 (IL-6), induced cell cycle arrest in the sub-G1 phase, and promoted caspase-dependent apoptosis in MCF-7/SC [33]. Both the mechanism of action and the results described by them indicate the potential antitumor activity of these saturated fatty acids. Our results suggest that genistein, depending on its size, influences the lipid metabolism of odd fatty acids. Its significantly higher concentration in animals fed with the nano- and microform may suggest a protective reaction in the rat organism in the form of fatty acids accumulation, which has a proven anticancer effect. However, it was found that, in the case of animals supplemented with genistein, there was an increase in the intensity of the carcinogenesis process. Considering the research results presented above, it can be assumed that the level of both fatty acids was too low to obtain anticancer effects. The results showed that fatty acids were increased within a physiological concentration range and not in extreme deficiency. These changes in fatty acid composition can be caused by other factors, such as excessive tumor growth or inhibition of certain enzymes. It is worth emphasizing that Bao’s experiment [33] was performed in cell models supplemented with high concentrations of fatty acids.

In our study, the MUFAs + PUFAs/SFAs ratio was calculated. Interestingly, this ratio was significantly the lowest in the control group of animals and highest in animals supplemented with micro- and nanogenistein. In two of her publications [34,35], Amézaga presented the results of studies that showed the concentration of saturated fatty acids to be lower in cancer patients than in the healthy control group. Since no cell can exist without membranes, the need to build new membranes is a priority in the process of cell replication. During the intensive replication of cancer cells, fatty acids are needed for their building, which means that their concentration and profile in the serum may be disturbed. In our study, the concentrations of individual MUFAs (C16:1 n-7, C16:1 n-9, C18:1 n-11) and PUFAs (C18:2 n-6 LA, C18:3 n-6 GLA, C18:3) n-3 ALA, C20:4 n-6 AA, C22:6 n-3 DHA) significantly increased in the groups of animals supplemented with nano- or microgenistein. Such an increase could be the result of carcinogenesis, not the cause. Earlier scientific studies clearly show that the concentrations of both MUFAs and PUFAs are higher in cancer patients than in control groups [21,31,32]. To meet the demands of rapid cell proliferation, cancer-related lipogenesis requires greater lipid production; therefore, de novo lipogenesis is believed to be an early and common phenomenon in the development of cancer. Saturated and monounsaturated fatty acids (SFAs and MUFAs) are part of de novo biosynthesis involving the enzyme complex fatty acid synthase (FASN) and delta-9-desaturase activity (∆9D, stearoyl-CoA desaturase, SCD-1); both of these enzymes are subject to overexpression in cancer [21]. In a study by Guo [32], the MUFAs/SFAs ratio was higher in the cancer group than that in the control group. These findings may suggest that de novo fatty acid synthesis has increased in the neoplastic area and that the cancer-related MUFA-to-PUFA conversion pathway may also be activated during cancer development [21]. Human cells, including cancer cells, cannot reproduce and grow without polyunsaturated fatty acids; therefore, the role they play is the subject of much debate and research. Depending on the type of tumor and its stage, tumor cells adjust their membrane properties to ensure their survival. PUFAs play important roles in the modulation of various processes such as metabolism, inflammation, cell signaling, and regulation of gene expression. The results from preclinical studies provide compelling evidence that PUFAs can mediate cancer progression in vitro and in vivo models of several different types of cancer. It was found that their levels are higher in oncological patients, such as in rats supplemented with nano and microgenistein [36]. Aberrant PUFA metabolism as a result of genetic variation may play a role in cancer risk and promote cancer progression. The metabolites most predominantly linked with cancer are leukotrienes and prostaglandins, as they have been shown to play important roles in the progression of cancer through angiogenesis, cell proliferation, metastasis, and apoptosis [36]. The results show a positive correlation between genistein supplementation and the activity of enzymes, in particular D5D. This indicates that a diet rich in genistein, especially in microparticles and nanoparticles in the case of D5D, accelerates the conversion of fatty acids. Both in animals supplemented with nano- and microgenistein, a statistically significant increase in the content of arachidonic acid in the serum of the animals was demonstrated. A similar relationship was not found for the EPA acid content in the serum. This indicates that a diet supplemented with genistein may increase enzyme activity, which will affect fatty acid conversion and eicosanoid synthesis. Increased levels of PGE2 have been found in several types of cancer, including in breast cancer [37]. As noted previously, PUFAs have pleotropic effects at the cellular and molecular levels. The associations between alterations in fatty acid profiles and metabolism and cancer risk and progression still require further research.

## 5. Conclusions

This is the first report that shows how genistein, as a nano-, microsensory, macromolecule, influences cholesterol and COP levels, as well as the composition and metabolism of fatty acids, in the serum of rats with neoplastic disease. This study found a negative effect of dietary supplementation with nanogenistein on the formation of cholesterol and oxysterols, which may have a pro-inflammatory effect.

## Figures and Tables

**Table 1:** Dynamic light scattering technique (DLS) results for obtained genistein particles [9].

DLS Parameter	Size (d nm) ^a^	Z-Average (d nm)	Zeta Potential ^b^ (mV)	Đ ^c^
Genistein nanoparticles	92 ± 41	158	−17.2 ± 5.5	1.000
Genistein microparticles	587 ± 83	1467	−30.2 ± 6.0	0.873

^a^—size ± standard deviation; ^b^—zeta potential ± standard deviation; ^c^—dispersity.

**Table 2:** Tumor induction based on supplementation.

Supplementation	Tumor Incidence (%) (Week 20)	The Week When First Tumor Occurred	Number of Tumors Per Animal (Week 20)	Tumor Weight (g) (Week 20) Mean ± SD	The Mean Number of Mitoses in the Field of View Area (Week 20) *	Tumor Grade
Standard	100% (8/8)	16 (2/8)	(2–9)	0.93 ± 1.34 (0.10–7.80) ^a^	1.79 ± 1.25 ^abc^	Adenocarcinoma 2 grade
Macrogenistein	100% (8/8)	17 (5/8)	(1–6)	1.27 ± 1.52 (0.14–6.39)	4.46 ± 2.38 ^ad^	Adenocarcinoma 2 grade
Microgenistein	88% (7/8)	17 (1/8)	(0–3)	1.99 ± 1.75 (0.11–6.11) ^a^	7.33 ± 1.57 ^bd^	Adenocarcinoma 3 grade
Nanogenistein	100% (8/8)	14 (1/8)	(2–5)	1.59 ± 2.64 (0.06–9.50)	5.82 ± 1.57 ^c^	Adenocarcinoma 3 grade

Data are expressed as mean ± SD. Values sharing letters indicate statistically significant differences between groups (*p* < 0.01); * Mitoses were counted in slides from randomly selected tumors in 15 fields of view with a 40× objective magnification.

**Table 3:** The animals’ body weight gain (g) (week 7–20).

Supplementation	The Animals’ Body Weight Gain (g) (Week 10–21)
standard	99.0 ± 10.9
macrogenistein	99.1 ± 8.8
microgenistein	107.4 ± 8.5
nanogenistein	96.5 ± 8.72

**Table 4:** Content of cholesterol, squalene, and oxysterols in serum of rats (μg/mL).

(μg/mL)	Standard	Macro	Micro	Nano	*p* Value
Squalene	19.79 ± 12.39	9.31 ± 2.90	10.53 ± 6.78	20.60 ± 13.54	n.s.
Cholesterol	2007 ± 1092 ^ab^	949 ± 259 ^a^	940 ± 425 ^a^	3643 ± 2727 ^b^	0.0213
7K-Ch	5.12 ± 2.60 ^ab^	2.64 ± 0.68 ^a^	3.05 ± 0.90 ^a^	8.04 ± 5.05 ^b^	0.012
7α-OH-Ch	1.81 ± 1.00 ^a^	1.10 ± 0.55 ^a^	0.88 ± 0.14 ^a^	3.57 ± 2.68 ^b^	0.017
7β-OH-Ch	4.69 ± 2.25	2.82 ± 0.52	3.17 ± 1.34	6.32 ± 4.7	n.s.
5,6βE-Ch	5.84 ± 3.03 ^a^	5.00 ± 0.48 ^a^	5.14 ± 0.91 ^a^	16.65 ± 11.99 ^b^	0.014
∑ COPs	17.45 ± 8.53 ^ab^	11.55 ± 1.18 ^a^	12.23 ± 2.87 ^a^	34.59 ± 24.16 ^b^	0.02
COPs/Ch (%)	0.94 ± 0.33	1.35 ± 0.56	1.68 ± 1.04	0.98 ± 0.14	n.s.

Data are presented as mean values ± standard deviation. ^a^, ^b^—homogeneous groups in rows (α = 0.05), n.s.—not significant; Standard—control group; Macro—a group of rats receiving genistein in macromolecules; Micro—group of rats receiving genistein in microparticles; Nano—a group of rats receiving genistein in nanoparticles; Σ COPs—sum of cholesterol oxidation products; Ch—cholesterol.

**Table 5:** FA profile in serum.

Fatty Acid (μg/mL)	Standard	Macro	Micro	Nano	*p* Value
**SFAs**					
C12:0 Lauric acid	12.57 ± 8.52	13.53 ± 3.43	16.24 ± 3.69	14.69 ± 5.27	n.s.
C14:0 Myristic acid	24.64 ± 25.68	18.28 ± 4.55	24.73 ± 3.82	26.85 ± 5.17	n.s.
C15:0 Pentadecanoic acid	13.58 ± 4.11 ^a^	15.32 ± 3.87 ^ab^	20.39 ± 5.09 ^bc^	23.64 ± 4.96 ^c^	0.0004
C16:0 Palmitic acid	617 ± 286	599 ± 144	736 ± 205	776 ± 118	n.s.
C17:0 Heptadecanoic acid	16.55 ± 3.55 ^a^	17.55 ± 2.81 ^ab^	21.97 ± 3.64 ^bc^	23.55 ± 4.60 ^c^	0.0015
C18:0 Stearic acid	359 ± 67	312 ± 55	412 ± 94	389 ± 71	n.s.
∑ SFAs	1043 ± 373	976 ± 199	1231 ± 299	1254 ± 193	n.s.
**MUFAs**					
C16:1 n-7 Palmitoleic acid	60.33 ± 13.61 ^a^	78.30 ± 15.47 ^ab^	102.31 ± 12.99 ^b^	98.13 ± 34.12 ^b^	0.0013
C16:1 n-9 9-Hexadecenoic acid	9.83 ± 3.10 ^a^	13.55 ± 3.42 ^a^	17.30 ± 4.25 ^b^	18.73 ± 4.37 ^b^	0.0003
C18:1 n-9 Oleic acid	230 ± 117	279 ± 77	338 ± 139	341 ± 89	n.s.
C18:1 n-11 Vaccenic acid	33.90 ± 10.94 ^a^	41.21 ± 9.51 ^ab^	53.65 ± 17.28 ^b^	57.91 ± 14.79 ^b^	0.0047
∑ MUFAs	334 ± 140	412 ± 95	511 ± 170	516 ± 137	n.s.
**PUFAs**					
C18:2 n-6 LA	588 ± 207 ^a^	676 ± 168 ^ab^	854 ± 241 ^ab^	861 ± 166 ^b^	0.023
C18:3 n-6 GLA	18.55 ± 5.07 ^a^	22.97 ± 6.08 ^ab^	30.20 ± 2.65 ^c^	27.79 ± 3.58 ^bc^	0.0001
C18:3 n-3 ALA	46.69 ± 15.30 ^ab^	37.01 ± 4.01 ^a^	51.78 ± 9.05 ^b^	53.91 ± 10.26 ^b^	0.015
C20:3 n-6 DGLA	6.30 ± 3.28	7.08 ± 2.25	7.49 ± 2.07	5.71 ± 1.96	n.s.
C20:4 n-6 AA	879 ± 167 ^ab^	824 ± 168 ^a^	1226 ± 197 ^c^	1114 ± 207 ^bc^	0.0004
C20:5 n-3 EPA	54.67 ± 24.89 ^b^	31.17 ± 7.81 ^a^	45.72 ± 10.28 ^ab^	50.72 ± 11.61 ^ab^	0.024
C22:6 n-3 DHA	170 ± 44.6 ^ab^	149 ± 30 ^a^	218 ± 52.5 ^b^	202 ± 36.3 ^ab^	0.0116
∑ PUFAs	1764 ± 410 ^a^	1748 ± 348 ^a^	2432 ± 412 ^b^	2314 ± 399 ^b^	0.0015

Data are presented as mean values ± standard deviation. ^a^, ^b^, ^c^—homogeneous groups in rows (α = 0.05), n.s.—not significant; Standard—control group; Macro—a group of rats receiving genistein in macromolecules; Micro—a group of rats receiving genistein in microparticles; Nano—a group of rats receiving genistein in nanoparticles; SFAs—saturated fatty acids; MUFAs—monounsaturated fatty acids; PUFAs—polyunsaturated fatty acids; Σ—sum.

**Table 6:** Fatty acid profile (%) in experimental groups.

Fatty Acids (μg/mL)	Standard	Macro	Micro	Nano	*p* Value
**SFAs**					
C12:0 Lauric acid	0.39 ± 0.19	0.45 ± 0.13	0.42 ± 0.18	0.36 ± 0.14	n.s.
C14:0 Myristic acid	0.70 ± 0.43	0.58 ± 0.07	0.61 ±0.14	0.66 ± 0.06	n.s.
C15:0 Pentadecanoic acid	0.43 ± 0.03 ^a^	0.48 ± 0.06 ^a^	0.49 ± 0.07 ^a^	0.58 ± 0.04 ^b^	0.0001
C16:0 Palmitic acid	19.0 ± 2.93	18.98 ± 1.87	17.40 ± 1.48	19.08 ± 1.18	n.s.
C17:0 Heptadecanoic acid	0.54 ± 0.09	0.57 ± 0.06	0.53 ± 0.06	0.58 ± 0.04	n.s.
C18:0 Stearic acid	11.77 ± 1.83 ^b^	10.03 ± 1.03 ^a^	9.84 ± 0.55 ^a^	9.53 ± 0.53 ^a^	0.0057
∑ SFAs	32.83 ± 2.04 ^b^	31.09 ± 1.27 ^ab^	29.28 ± 1.35 ^a^	30.78 ± 0.89 ^a^	0.0005
**MUFAs**					
C16:1 n-7 Palmitoleic acid	0.31 ± 0.03 ^a^	0.43 ± 0.06 ^b^	0.41 ± 0.05 ^b^	0.46 ± 0.06 ^b^	0.0001.
C16:1 n-9 9-Hexadecenoic acid	1.97 ± 0.40	2.56 ± 0.58	2.50 ± 0.33	2.37 ± 0.75	n.s.
C18:1 n-9 Oleic acid	7.04 ± 1.38	8.80 ± 1.22	7.82 ± 1.93	8.26 ± 1.11	n.s.
C18:1 n-11 Vaccenic acid	1.08 ± 0.15 ^a^	1.31 ± 0.15 ^b^	1.26 ± 0.16 ^ab^	1.40 ± 0.18 ^b^	0.0028
∑ MUFAs	10.40 ± 1.33 ^a^	13.10 ± 1.39 ^b^	12.00 ± 1.91 ^ab^	12.48 ± 1.93 ^ab^	0.0201
**PUFAs**					
C18:2 n-6 LA	18.49 ± 1.71 ^a^	21.41 ± 2.19 ^b^	20.18 ± 1.89 ^ab^	21.04 ± 1.07 ^b^	0.0125
C18:3 n-6 GLA	0.60 ± 0.12	0.76 ± 0.25	0.75 ± 0.17	0.68 ± 0.08	n.s.
C18:3 n-3 ALA	1.49 ± 0.25 ^b^	1.21 ± 0.23 ^a^	1.25 ± 0.05 ^ab^	1.32 ± 0.16 ^ab^	0.0297
C20:3 n-6 DGLA	0.19 ± 0.06	0.23 ± 0.07	0.19 ± 0.07	0.14 ± 0.06	n.s.
C20:4 n-6 AA	28.70 ± 3.86	26.42 ± 3.20	29.83 ± 4.20	27.32 ± 2.48	n.s.
C20:5 n-3 EPA	1.81 ± 0.87 ^b^	1.01 ± 0.21 ^a^	1.16 ± 0.42 ^ab^	1.24 ± 0.18 ^ab^	0.0185
C22:6 n-3 DHA	5.46 ± 0.66	4.76 ± 0.34	5.34 ± 1.65	4.97 ± 0.54	n.s.
∑ PUFAs	56.76 ± 3.15	55.80 ± 2.20	58.71 ± 3.08	56.73 ± 1.84	n.s.
(MUFAs + PUFAs)/SFAs	2.05 ± 0.17 ^a^	2.22 ± 0.13 ^ab^	2.42 ± 0.16 ^c^	2.25 ± 0.09 ^bc^	0.0003

Data are presented as mean values ± standard deviation. ^a^, ^b^, ^c^—homogeneous groups in rows (α = 0.05), n.s.—not significant; Standard—control group; Macro—a group of rats receiving genistein in macromolecules; Micro—a group of rats receiving genistein in microparticles; Nano—a group of rats receiving genistein in nanoparticles; SFAs—saturated fatty acids; MUFAs—monounsaturated fatty acids; PUFAs—polyunsaturated fatty acids; Σ—sum.

## Data Availability

The datasets generated for this study are available on request to the corresponding author.
